# DYADIC LONELINESS AND COGNITIVE HEALTH AMONG OLDER MARRIED COUPLES: LONGITUDINAL EVIDENCE FROM THE HRS

**DOI:** 10.1093/geroni/igac059.465

**Published:** 2022-12-20

**Authors:** Jeffrey Stokes, Anyah Prasad, Adrita Barooah

**Affiliations:** University of Massachusetts Boston, Boston, Massachusetts, United States; University of Massachusetts Boston, Boston, Massachusetts, United States; University of Massachusetts Boston, Boston, Massachusetts, United States

## Abstract

Loneliness is associated with numerous poor health outcomes, including mortality. Additionally, loneliness is not merely an isolated individual’s experience; rather, loneliness occurs regularly even among the married, and can affect both spouses’ health. We analyze 3-wave dyadic data from the Health and Retirement Study (2008-2018; N = 7,907 dyads) to determine (a) whether loneliness is associated with participants’ own and/or their partner’s verbal fluency and episodic memory over a nearly decade-long period, and (b) whether these measures of cognitive health predict older spouses’ own or their partner’s loneliness over the same period. Results indicated that (1) loneliness, episodic memory, and verbal fluency were all “contagious” within couples, such that baseline levels of each predicted participants’ own and their partner’s values at follow-up; (2) participants’ own loneliness was associated with poorer verbal fluency and episodic memory at follow-up; and (3) neither participants’ own nor their partner’s cognitive functioning predicted future loneliness.

